# Facial and mandibular landmark tracking with habitual head posture estimation using linear and fiducial markers

**DOI:** 10.1049/htl2.12076

**Published:** 2024-02-05

**Authors:** Farhan Hasin Saad, Taseef Hasan Farook, Saif Ahmed, Yang Zhao, Zhibin Liao, Johan W. Verjans, James Dudley

**Affiliations:** ^1^ Department of Electrical and Computer Engineering North South University Dhaka Bangladesh; ^2^ Adelaide Dental School University of Adelaide Adelaide Australia; ^3^ Department of Computer Science and Information Technology La Trobe University Melbourne Australia; ^4^ Australian Institute for Machine Learning University of Adelaide Adelaide Australia

**Keywords:** image processing, motion estimation

## Abstract

This study compared the accuracy of facial landmark measurements using deep learning‐based fiducial marker (FM) and arbitrary width reference (AWR) approaches. It quantitatively analysed mandibular hard and soft tissue lateral excursions and head tilting from consumer camera footage of 37 participants. A custom deep learning system recognised facial landmarks for measuring head tilt and mandibular lateral excursions. Circular fiducial markers (FM) and inter‐zygion measurements (AWR) were validated against physical measurements using electrognathography and electronic rulers. Results showed notable differences in lower and mid‐face estimations for both FM and AWR compared to physical measurements. The study also demonstrated the comparability of both approaches in assessing lateral movement, though fiducial markers exhibited variability in mid‐face and lower face parameter assessments. Regardless of the technique applied, hard tissue movement was typically seen to be 30% less than soft tissue among the participants. Additionally, a significant number of participants consistently displayed a 5 to 10° head tilt.

## Graphical abstract

Use of deep‐learning based facial landmark tracking to monitor jaw movements and habitual head posture.

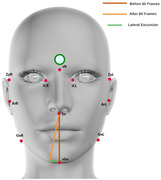

## 1 INTRODUCTION

Habitual head posture varies among individuals and has been shown to influence functional jaw movement [1]. While maximum mouth opening can be measured through various methods such as still photography and electronic rulers, objectively measuring lateral excursions without specialised devices poses a challenge [1]. Lateral jaw movements exhibit individual variability, and using the destabilised soft tissue of the chin as a reference point for jaw movement measurement has proven unreliable. This unreliability is particularly notable when habitual head tilting introduces an additional source of variation [2].

Technique‐sensitive devices like electrognathography [3, 4] and optoelectronic tracking [5] do not focus on soft tissue mandibular movement [1]. Monitoring such movement, however, is now important with increased global applications of botulinum injections for cosmetic and rehabilitative purposes [6] and the recent research interest in studying facial neuropathies and paralyses [7]. Historically, soft tissue movement has been tracked either arbitrarily by measuring anatomical distances between two fixed landmarks or through fiducial markers of a known diameter affixed onto the participant [8]. The current study is the first to document and compare both methods in evaluating mandibular movements on the lateral plane within a South Australian population.

The current research aimed to achieve two main objectives: firstly, to assess the precision of facial feature measurements using fiducial markers versus arbitrary facial width measurements, and secondly, to provide a quantitative evaluation of the lateral soft tissue movement of the mandible relative to the hard tissue movement, and to detect head tilts using consumer‐grade camera videos. To achieve both objectives, the study made modifications to an in‐house open access software application that was designed to detect distances between facial landmarks by converting pixels to millimetres through object detection models such as OpenCV and Dlib [9, 10].

It was hypothesised that no significant discrepancies would be observed between the two estimation techniques when purposed to measure facial parameters during jaw movement on the lateral plane.

## 2 METHODOLOGY

### 2.1 Study design

The study was approved by the University of Adelaide Human Research and Ethics Committee (H‐2022‐185).

### 2.2 Development of the tracking software

The developed software comprises of Dlib face detector model for the frontal face detection and FAN's face alignment model for the side face detection and is an adaptation of the Dental Loop FLT model [10]. Dlib, a C++‐based library, was utilised along with deep neural networks ResNet designed for facial landmark recognition. The facial detection technique employed by Dlib makes use of histogram‐oriented methods (HOG). This approach, with certain adaptations such as single face focused detection, was integrated to identify facial landmarks and outlines. To adapt ResNet for the specific task of facial landmark recognition, transfer learning techniques were utilised on the dataset [11]. These tasks were accomplished through a pretrained model referred to as ‘shape_predictor_68_face_landmarks.dat’. The shape_predictor_68_face_landmarks.dat file contains the weights and architecture of a trained deep learning model (typically a shape predictor based on regression trees) that has been trained to predict 68 specific landmarks on a human face. The study also utilised the 2D ‘Face Alignment’ algorithm extracted from the face alignment network (FAN) model [12]. It is a convolutional neural network‐based approach for predicting the positions of facial landmarks, allowing for alignment and analysis of facial features in 2D images. This choice was made to address the limitations of Dlib in reliably detecting faces that are obscured or positioned at angles.

**FIGURE 1:**
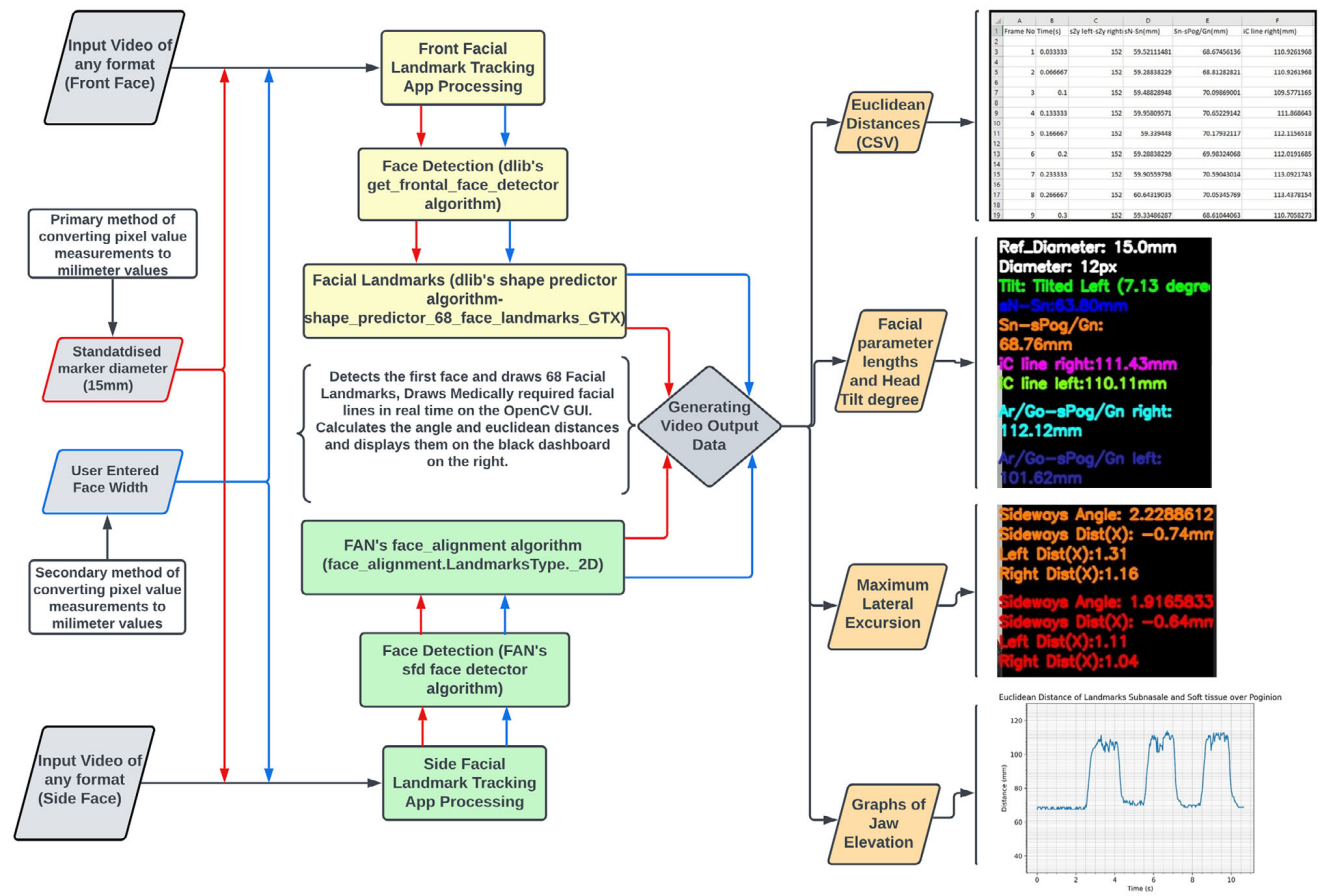
Workflow of development.

To enable the detection of facial features from within images or frames, an algorithm was developed using Dlib's ‘get_frontal_face_detector()’ method which detects a frontal face and returns the landmarks of the detected face. Additionally, an extra memory functionality was implemented. This feature enhanced reliability and maintained focus on a singular face within the frame consistently during a recording session for continuous tracking. Within a continuous loop, every frame of the video was extracted and transformed into a grayscale representation. The grayscale image was then subjected to loaded landmark and face detection models, and display values for corresponding landmarks. A novel reference point to highlight the soft tissue over nasion (sN) was also implemented. The iterative procedure was set to terminate following completion of processing of all frames. The workflow has been reported below in Figure 1.

### 2.3 Development of the circular fiducial marker approach

The circular fiducial marker approach involved the use of the Hough circle transform (HoughCircles) function from OpenCV to identify a standardised circular marker placed strategically on the participants' foreheads or necks, depending on the orientation of the face being detected. The Hough circle transform is a technique used for detecting circular shapes within an image, even when they may be partially obscured or vary in size or position [9]. The marker's diameter was then computed based on its pixel‐based representation. A predefined region of interest (ROI), determined by the locations of eyebrow landmarks, encapsulates the marker for detection. The marker's contrast against the skin tone ensured reliable identification, irrespective of colour variation.

The conversion from pixel‐based measurements to millimetres was achieved through a customisable reference marker diameter, which was standardised to 15 mm commercially available markers in this study. This was determined following trial and error to determine the dimension The conversion rate was computed as follows:

$$\mathrm{Conversion}\;\mathrm{Rate}\;=\;\frac{\mathrm{Reference}\;\mathrm{Circle}\;\mathrm{Diameter}\;\left(\mathrm{mm}\right)}{\mathrm{Detected}\;\mathrm{Circle}\;\mathrm{Diameter}\;\left(\mathrm{Pixel}\right)}$$

In this equation, the reference circle diameter represents the known physical diameter of the circular fiducial marker in millimetres, while the detected circle diameter corresponds to the measured diameter of the marker in pixels within the image.

### 2.4 Development of arbitrary width reference approach

**FIGURE 2:**
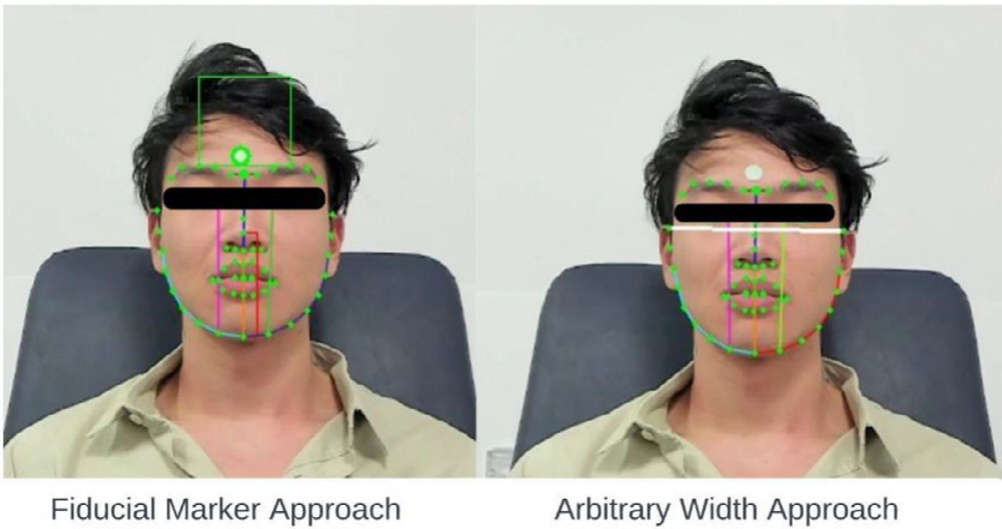
Visual representation of the Fiducial marker approach versus the arbitrary width reference approach.

In the arbitrary width reference approach, the inter‐zygion reference was considered and labelled: ‘sZy left—sZy right’ which denoted the facial width between the soft tissue over the zygomatic prominence. The pixel‐based measurements along said lines corresponded to the detected face width measurements obtained from the multi‐step process outlined earlier. Figure 2 demonstrates live examples of the two approaches. The pixel‐based measurements were then converted to millimetres using the formula:

$$\begin{array}{ccc} & & A\mathrm{natomical}\;\mathrm{Distance}\;\mathrm{in}\;\mathrm{millimetre}\\ & & =\;\frac{\mathrm{Distance}\;\mathrm{in}\;\mathrm{Pixel}}{\mathrm{Reference}\;\mathrm{in}\;\mathrm{Pixel}}\;\times \;\mathrm{User}\;\mathrm{provided}\;\mathrm{reference}\;\mathrm{in}\;\mathrm{millimetre} \end{array}$$

### 2.5 Landmark tracing

The landmarks registered for the purpose of current evaluation were: (1) distance from soft tissue over nasion to subnasale, (2) distance from subnasale to soft tissue over pogonion, (3) left and right inner Canthus lines, (4) distance from articularis to gonion to soft tissue over gnathion on the right and left sides, (5) distance between the soft tissue over right and left zygion. The order of landmarks and corresponding facial features have been adapted from the open‐source software Dental Loop FLT [10].

### 2.6 Quantifying mandibular lateral excursions and head tilt

To track lateral excursion, a reference line from subnasale to soft tissue over gnathion was established. To account for limitations in computational power, changes in parameters were automatically recorded at 60 frame intervals from 30 fps 1080p video recordings.

**FIGURE 3:**
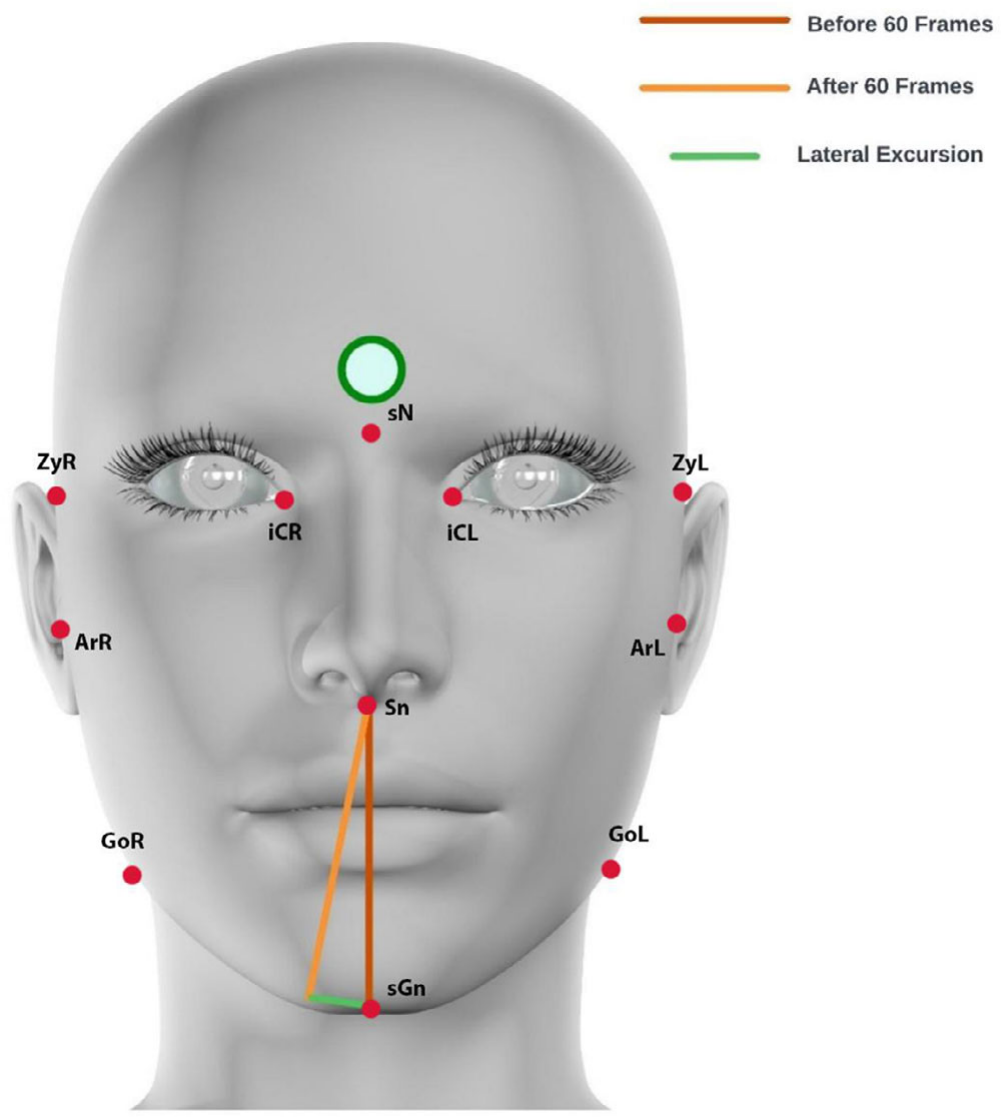
Visual representation of the landmarks and quantification of lateral excursion across 60 frames. (ZyR; Soft tissue over right zygomaticus, ZyL; Soft tissue over left zygomaticus, sN; Soft tissue over nasion, iCR; Inner canthus right, iCL; Inner canthus left, ArR; Soft tissue over right articularis, ArL; Soft tissue over left articularis, GoR; Right gonion, GoL; Left gonion, sGn; Soft tissue over gnathion, Sn; soft tissue over subnasale).

**FIGURE 4:**
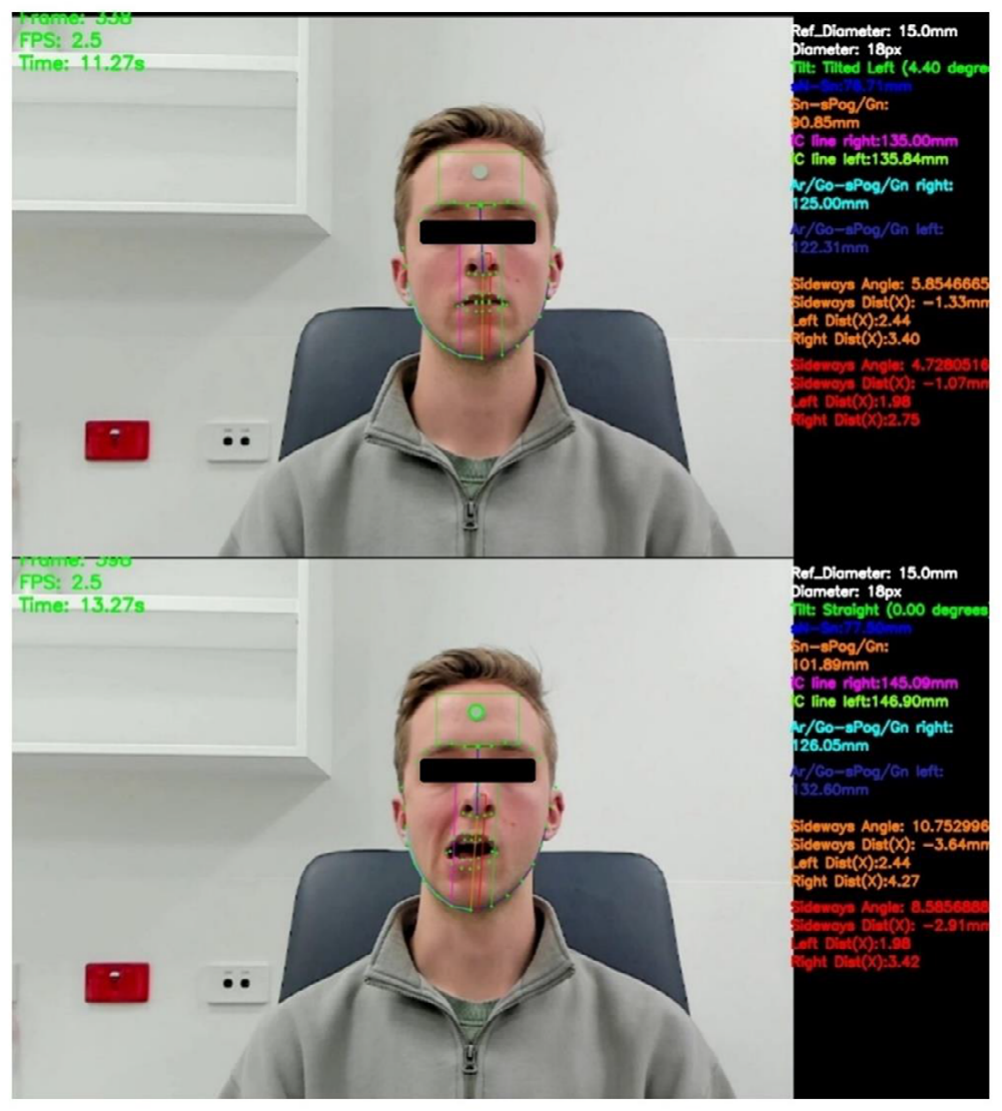
Recording of vertical and horizontal displacements at the time of lateral excursion to exclude movement anomalies.

The primary goal of this tracking algorithm was to quantify the angular extent of the jaw's lateral movement. To achieve this, the algorithm calculated the angle between vectors that represented the initial and final directions of movement (Figure 3). This angle, measured in radians, served as an indicator of the magnitude of the jaw's lateral excursion displacement. Moreover, the algorithm also accounted for vertical and horizontal displacements concerning the specific chin landmark at the time of lateral excursion to exclude movement anomalies (Figure 4).

### 2.7 Head tilt

**FIGURE 5:**
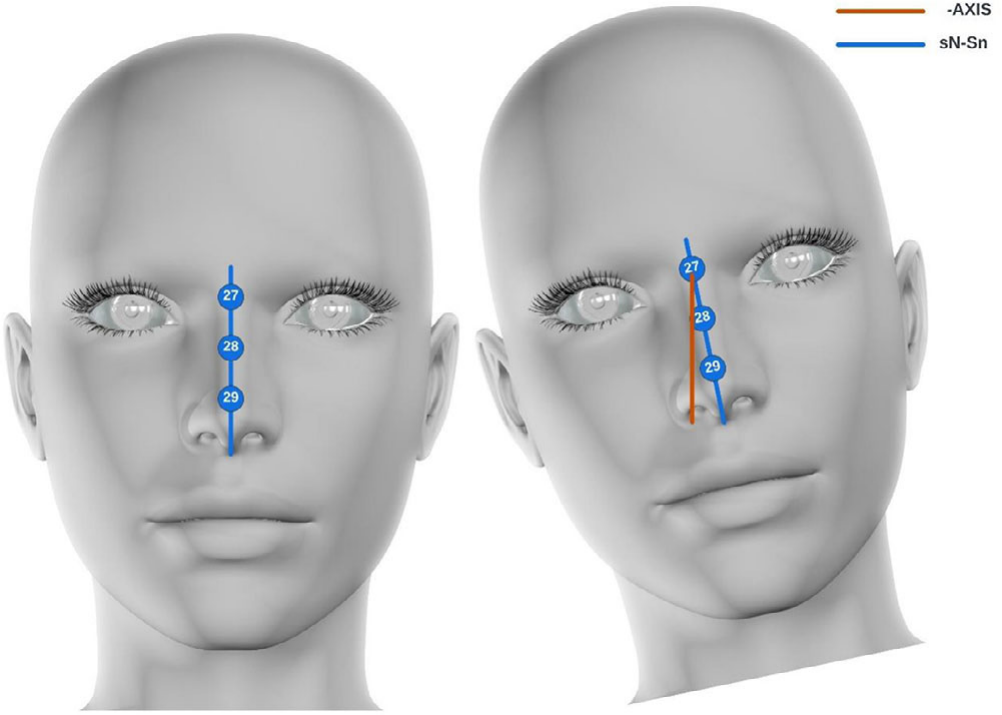
The custom landmarks and determination of direction of tilting.

**FIGURE 6:**
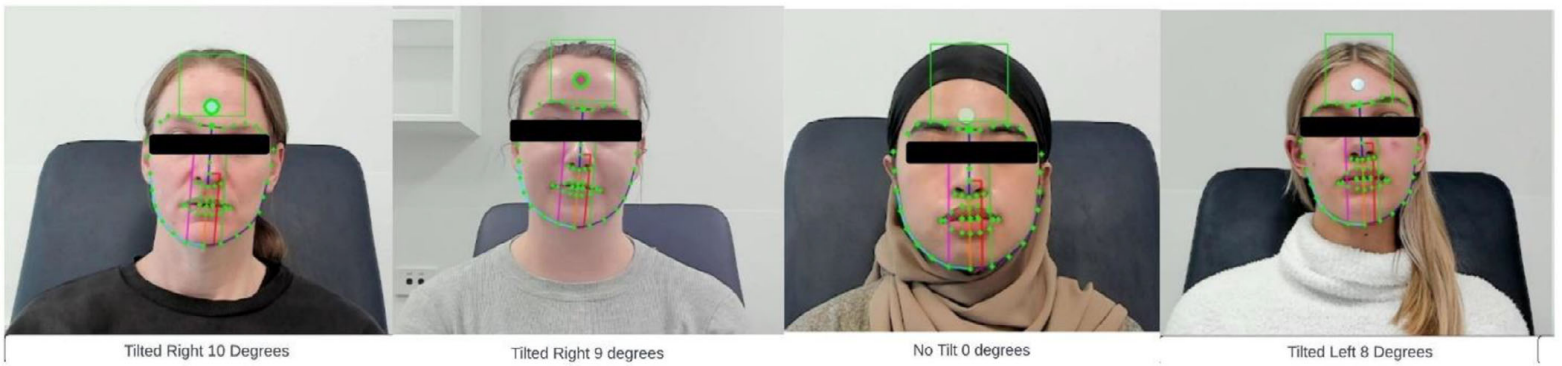
Clinical demonstration of the detection of degree and direction in head tilting.

To detect head tilt, a dedicated function, “calculate head tilt,” was defined. The function began by extracting Landmarks 27 and 29 from the Dlib model and computing the horizontal distance between them. To provide a reference point for this measurement, the function also computed the distance between the top nose landmark and the middle nose landmark (Figure 5). Thresholds were adjusted, and the directional angle of tilting was then calculated using the ‘math.atan’ function and differentiated by positive and negative values to indicate the direction of tilt. This determination was made based on the sign of the horizontal distance: a positive sign denoted a tilt to one direction, while a negative sign indicated a tilt in the opposite direction (Figure 6).

### 2.8 Data collection

Based on a large effect size of *F* = 0.55 (*G* × Power, JPD paper), *α* = 0.05 and power of 0.80, it was determined that data from 37 participants would be required to make the comparisons. Thirty‐seven adult participants aged between 18 and 65 with some or all their natural dentition and without temporo‐mandibular dysfunction were recruited. The participants undertook a single session of video recording using a consumer camera (Brio‐4K, Logitech, USA) at 1080p at 60 fps using a 13‐megapixel lens with no ambient lighting regulation. Video outputs were at 2500 Kbps native bitrate and encoded using H.264 NVENC and exported in Matroska Video (.mkv) format. In the clinic where data was collected, participants were uniformly positioned 45 cm away from the lens of a camera fixed on a tripod mount. This setup was carefully chosen for optimal focus in the prevailing lighting conditions. All data that could be used to identify subjects was anonymised, and recordings were deidentified in compliance with the guidelines provided by the university's human research ethics committee. Measurements for inter‐zygion facial width, lower face height (from subnasale to the soft tissue over gnathion), and midface height (from the soft tissue above nasion to the soft tissue over subnasale) were taken with a measuring ruler by a single operator, establishing the baseline for reference tracking. The hard tissue measurements for lateral excursion were captured using an electrognathograph (JT‐3D; BioResearch, USA) to establish comparative differences between hard and soft tissue movement. Before data collection, a second operator conducted ten independent readings to confirm the reproducibility and repeatability of measurements. This process resulted in an Intraclass correlation coefficient (ICC) of 0.86 and an average variation of 1.5 mm. Intra‐operator reliability was also assessed with five independent readings across two consecutive days, yielding an ICC of 0.89.

### 2.9 Data analysis

The data derived from the circular fiducial marker approach and the arbitrary face width reference approach were compared with the physically measured values estimated by the operator on the participants at the time of video recording. The discrepancies between the methods' measurements and the ground truth values, taking into consideration a margin of error, were quantitatively analysed as relative and absolute errors. Test for normality was carried out by the Kolmogorov‐Smirnov test and a 1‐way ANOVA was performed at multiple levels to compare across the different methods in tracking facial parameters and mandibular lateral excursions. The following formula were used in the analyses of data:

$$F-\mathrm{statistic},\;F=\frac{\mathrm{Explained}\;\mathrm{Variance}/\mathrm{Degrees}\;\mathrm{of}\;\mathrm{Freedom}\;}{\mathrm{Residual}\;\mathrm{Variance}/\mathrm{Degrees}\;\mathrm{of}\;\mathrm{freedom}\;}$$

$$\mathrm{Relative}\;\mathrm{Error},\;\;RE\;=\frac{\mathrm{Observed}\;\mathrm{Value}\;-\;\mathrm{True}\;\mathrm{Value}}{\mathrm{True}\;\mathrm{Value}}$$

$$\mathrm{Absolute}\;\mathrm{Error},\;\;AE\;=\;\left|\mathrm{Observed}\;\mathrm{Value}-\mathrm{True}\;\mathrm{Value}\right|$$

$$p-\mathrm{Value},\;z\;=\frac{\overset{\wedge }{p}-p0}{\sqrt{\frac{po\left(1-p0\right)}{n}}}$$

## 3 RESULTS

**FIGURE 7:**
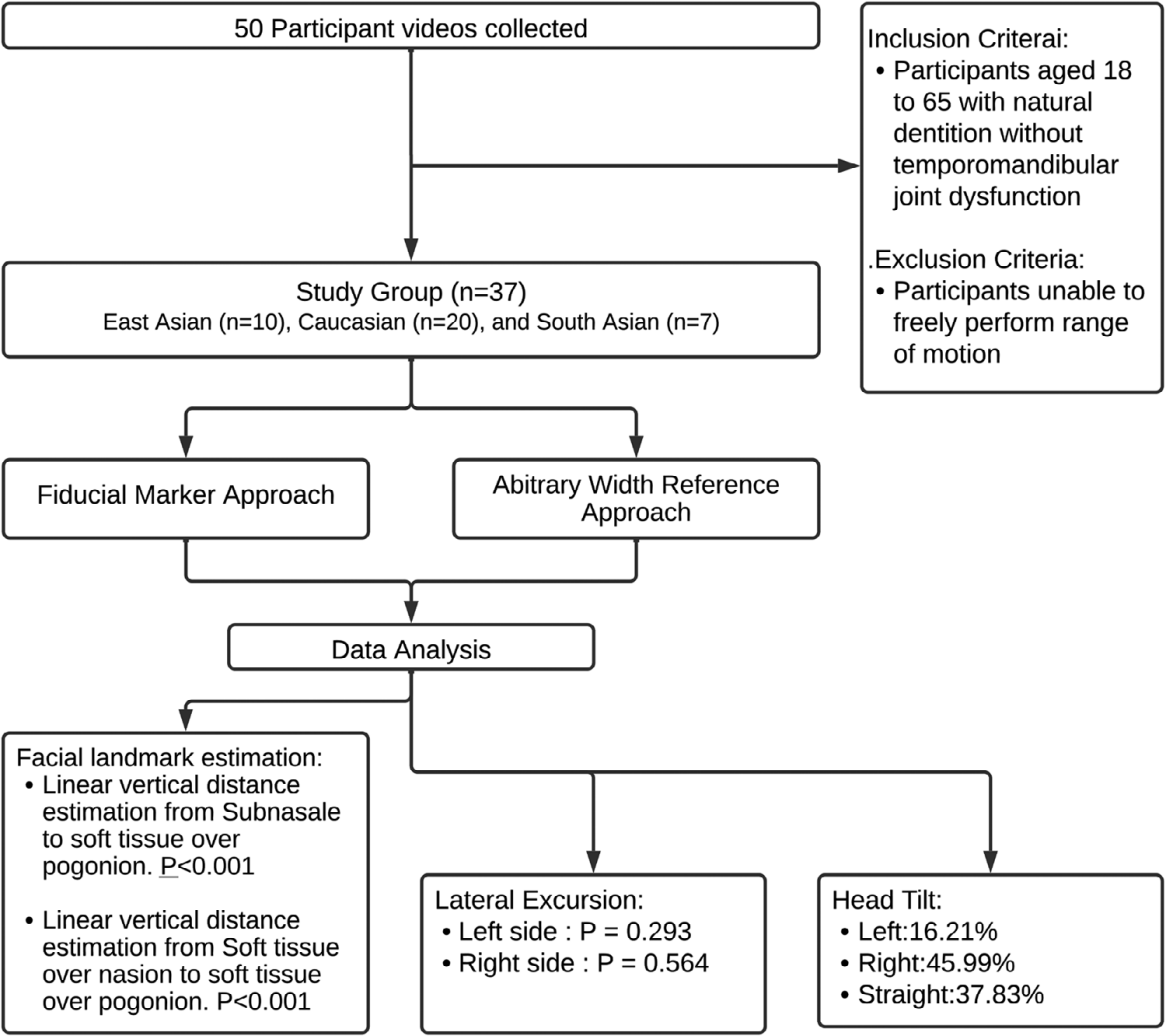
Summary flowchart of the process workflow.

Data was collected from a sample of 37 participants, spanning three distinct demographic groups: East Asian (*n* = 10), Caucasian (*n* = 20), and South Asian (*n* = 7). Of these, 12 were male and 25 females. Fourteen participants demonstrated a habitual head tilt between 0° and 10° to the right, six demonstrated a head tilt of same degrees to the left, three tilted 10–15° to the right, while the remaining 14 maintained a straight posture. The methodology for data collection and synthesis is illustrated in Figure 7. Both the relative and absolute errors of the two tracking methods across the participant pool are detailed in Table 1.

**TABLE 1:** Tracking method absolute and relative error.

	Arbitrary width reference approach				Fiducial tracking approach			
	Lower face		Mid face		Lower face		Midface	
	Relative	Absolute	Relative	Absolute	Relative	Absolute	Relative	Absolute
1	9.39	6.57	19.64	22	10.99	7.69	25	28
2	0.44	0.3	3.23	4.33	0.06	0.04	0	0
3	5.65	3.73	8.94	11.09	2.88	1.9	2.61	3.24
4	4.94	3.46	8.16	9.63	4.84	3.39	9.14	10.79
5	3.26	2.35	2.76	3.59	4.51	3.25	0.25	0.32
6	1.24	0.82	7.95	9.86	0.79	0.52	14.52	18
7	5.21	3.65	3.56	4.63	9.36	6.55	10.54	13.7
8	6.05	4.48	7.62	9.9	11.82	8.75	1.88	2.44
9	4.04	2.75	12.07	14.97	0.44	0.3	11.05	13.7
10	4.1	3.28	5.78	8.09	1.63	1.3	2.03	2.84
11	13.75	11.55	0.75	0.97	3.21	2.7	13.29	17.28
12	11.37	8.87	5.62	7.53	0.64	0.5	6.62	8.87
13	8.54	5.98	2.61	3.13	7.99	5.59	16.88	20.26
14	10.14	7.1	5.19	6.23	10.94	7.66	4.78	5.74
15	5.24	3.67	3.46	4.33	2.59	1.81	12.26	15.32
16	10.83	7.8	3.38	4.23	4.86	3.5	10.95	13.69
17	3.58	2.58	5.68	7.95	8.06	5.8	15.57	21.8
18	19.25	16.36	2.65	3.6	6.12	5.2	8.82	12
19	0.4	0.28	5.2	6.45	10.29	7.2	15.05	18.66
20	21.06	18.95	5.17	7.5	11.11	10	6.21	9
21	12.3	9.35	0.42	0.54	5.07	3.85	5.73	7.45
22	13.3	10.91	0.88	1.158	16.93	13.88	3.04	4.01
23	17.11	13	1.77	2.3	17.11	13	2.06	2.68
24	35.8	28.64	14.93	19.11	25	20	5.47	7
25	20.33	18.3	5.71	8.28	5.21	4.69	13.34	19.35
26	27	21.6	8.55	11.11	23.29	18.63	4.28	5.57
27	17.6	14.96	5.74	7.69	11.76	10	12.01	16.1
28	18.88	16.05	6.04	8.34	12.25	10.41	2.64	3.65
29	12.34	10.49	0.44	0.61	18.82	16	0.09	0.12
30	11.14	9.47	2	2.76	4.52	3.84	9.19	12.68
31	14.44	10.83	3.41	4.16	7.71	5.78	11.75	14.34
32	23.44	16.88	11.85	16.12	12.6	9.07	4.92	6.69
33	15.97	14.37	7.15	10.58	11.11	10	0.28	0.41
34	26.86	24.17	9.65	14.28	12.22	11	0.87	1.29
35	1.83	1.43	0.4	0.55	3.06	2.39	8.55	11.8
36	47.43	47.43	24.17	35.77	38.98	38.98	17.02	25.19
37	16.98	13.58	8.29	11.27	3.41	2.73	13.46	18.31

**FIGURE 8:**
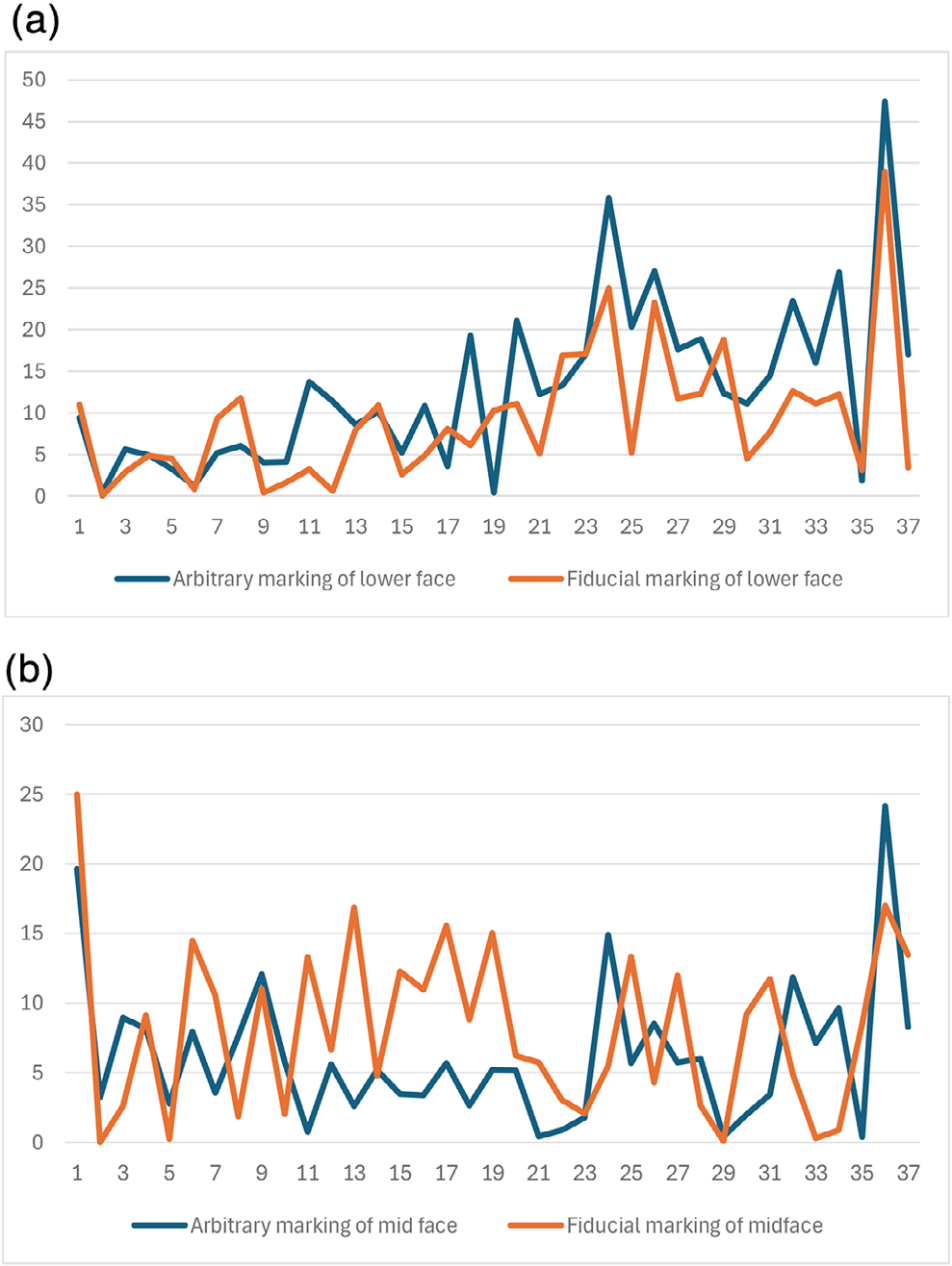
(A) Relative errors in the two estimation methods for lower face tracking. (B) Relative errors in the two estimation methods for mid face tracking.

The relative error trends across 37 participants for lower face (Figure 8A) and mid face estimation (Figure 8B) were then visualised. Higher errors were observed in a few instances and have been discussed in the following section.

When compared to physical measurements, there were notable differences across measurement techniques. Particularly, using FM resulted in a higher standard deviation in estimating lower face height. (Table 2).

**TABLE 2:** Evaluation of the three soft tissue landmarks using different tracking techniques.

	Mean	SD	MSE	F‐stat	P‐value
**Linear vertical distance to estimate lower face height**					
Physical values from arbitrary measurement	77.67	8.83		16.13	<0.001
Detected value from arbitrary width reference approach	67.99	7.05	201.09		
Detected value from Fiducial Marker tracking	71.78	31.17	108.80		
**Linear vertical distance to estimate mid face height**					
Physical value from arbitrary measurement	132.05	8.33		10.61	<0.001
Detected value from arbitrary width reference approach	130.43	10.36	115.00		
Detected value from Fiducial Marker tracking	139.69	10.55	168.74		

* Significant < 0.05; 1‐way ANOVA; SD, standard deviation; MSE, mean squared error.

The mean electrognathograph values were 8.85 ± 2.41 on the left side while it was 8.97 ± 2.10 on the right side and were approximately 30% less than soft tissue estimations performed by both AWR and FM techniques (Table 3).

**TABLE 3:** Measurements of maximum lateral excursion and their corresponding electrognathograph measurements on the left and right side.

	Left			Right		
Participant	Electro‐gnathograph	AWR	FM	Electro‐gnathograph	AWR	FM
1	5.3	7.09	7.6	5.4	3.42	3.7
2	3.7	5.29	6.36	10.6	18.53	19.16
3	5.2	2.30	3.44	5.3	3.15	4.74
4	9	11.36	15.92	6	4.18	4.13
5	10.2	5.6	6.06	10	5.02	5.45
6	7.9	6.03	6.88	7.3	2.5	2.26
7	12.7	12.5	15.62	8.1	10.24	12.2
8	7.9	1.8	1.97	8.6	3	3.68
9	8.5	6.7	8.96	10.3	7.8	8.81
10	10.2	2.5	3.39	6.6	2.5	2.92
11	11	2.7	3.59	10.6	3.8	4.83
12	10.7	4.9	5.6	13.2	4.6	5.14
13	7.7	2	2.19	9.4	3.02	3.96
14	8.2	3.25	3.74	9.4	2.55	2.66
15	11.8	3.20	3.63	10.2	5.34	5.76
16	9.4	5.5	6.87	7.7	4.22	4.42
17	9.4	4.6	5.96	5	12.89	13.27
18	10.8	8.9	10.96	10.4	16.7	17.86
19	10.3	1.34	1.56	8.8	4.56	4.76
20	8.4	6.74	7.32	8.8	6.79	7.32
21	10.4	3.44	4.77	12.3	8.89	9.91
22	8.4	4.53	5.34	10.7	4.56	5.44
23	9.7	2.67	3.81	8.4	7.21	7.44
24	9.4	3.22	4.24	8.2	3.01	3.29
25	1.3	6.44	7.43	4.1	4.11	4.19
26	7.2	8.33	10.27	9	16.6	18.5
27	9.1	3	3.16	10.1	5.21	5.43
28	9.4	3.4	5.36	10.2	3.41	4.25
29	12	9.1	12.15	12.9	5.67	6.02
30	7.4	6.79	9.36	8.6	5.89	6.28
31	8	4	4.47	9.1	2.56	2.09
32	8.6	3.9	4.69	9.3	3.55	4.13
33	7.8	3.12	3.27	9.5	3.04	3.03
34	9.2	2.32	2.82	11.3	3.2	3.3
35	9	6.5	7.33	10.1	5.6	6.35
36	7.7	2.3	3.31	7.3	3.21	3.28
37	14.5	15.2	19.19	8.9	6.75	8.65

## 4 DISCUSSION

The initial hypothesis anticipated minimal disparities between the two estimation techniques when applied to facial parameters or during lateral movement monitoring. However, when comparing the estimated values to the physical arbitrary measurements, notable discrepancies arose, prompting the rejection of our initial hypothesis. Standard deviations and means for lateral excursions on both sides showed resemblance between the two measurement techniques. This suggests reasonably consistent results within groups with variability potentially arising from external conditions and population‐specific lateral excursion patterns [13].

It was observed that East Asians exhibited a slightly higher mean head tilt angle (9.133° ± °1.99°) compared to Caucasians (7.375° ± °2.05°). The findings become more generalised and relevant as head tilting was observed in 62.2% of the current dataset and across ethnicities. Habitual head posture is an important determinant of jaw function and in some cases can predispose to long term masticatory muscle complex dysfunction. While superior to the alternative method of using traditional goniometers to measure habitual head postures [1], an objective analysis that accounts for head tilting similar to current methods can be of limited value in individuals with undiagnosed neck pathologies such as cervical and spinal injuries that demonstrate compensatory tilting mechanisms [14].

### 4.1 Limitations of the two tracking systems

Each tracking method demonstrated inherent limitations, necessitating a thorough understanding of the shortcomings for accurate result interpretation.

The fiducial marker approach exhibited higher variability in estimating mid‐face height, attributed to its dependence on the Hough circle detector. The Hough circle detector is a specific image processing technique used to detect circles within a given image. In some cases, discrepancies arose when the contrast between the circle's background and perimeter did not meet expected criteria, causing fluctuating output values while in other instances, hair over the forehead and makeup residues contributed to occluded‐angle images and noise [15]. Notably, using physical arbitrary measurements from a single operator as a standard may have introduced some observer‐generated biases and parallax errors.

Environmental factors, such as lighting and electromagnetic interference, can impact tracking systems variably, influencing readings. Although existing datasets had participants in suitable ambient lighting conditions, fiducial markers, in some instances, captured excessive skin surface reflections, causing momentary disruptions in data generation as the system temporarily lost track. While the arbitrary width reference approach is less susceptible to skin surface reflections, it is more vulnerable to sudden head movements.

### 4.2 Limitations in the image acquisition process

There were several complexities associated with determining the duration a participant took to complete a cycle of lateral excursions. As this process was subjective, variations were noted across different ethnic groups as which may have significant correlation with head tilting. This variability was further compounded by sporadic, unanticipated head movements and occasional jittery motions that the participants performed during the data collection process. Consequently, these occasional irregularities necessitated manual frame‐by‐frame scrutiny to ensure the precision and fidelity of data retrieval from the recorded sequences thereby preventing the workflow from becoming a fully unsupervised process.

The current model overzealously considered every frame and interval while continuously updating the generated report to the highest value, occasionally collecting irrelevant data due to unintended movements during facial expressions like smirking. The current dataset's native sampling rate of 60 fps was deemed sufficient for capturing lateral movement values, although anatomical variations in the temporomandibular joint complex could have contributed to higher overall estimation [16, 17]. To address some of these limitations, the software correlated movement with habitual head posture orientation through threshold adjustments. A similar technique was used by Hussein et al. for iris pattern recognition in ocular torsional changes due to head tilt [18].

### 4.3 Future recommendations

Attempting to create prediction models for jaw movement trends without accurately identifying habitual head postures may result in overfitting and inaccuracies. A potential solution is to gather repeated data from the same individual over several weeks, identifying trends in habitual head posture changes and fitting a model through clustering recurring patterns. This approach could be a focus for future research. Notably, deep learning‐based time‐series experiments at the Royal Adelaide Hospital in South Australia are already exploring trends influencing patient discharge [19].

In future software implementations, the utilisation of models like Anchorface, capable of capturing occluded faces, can enable the analysis of lateral excursions in the sagittal plane and anterior protrusion in the coronal plane from video recordings at obscured angles. This holds clinical significance in evaluating temporomandibular joint conditions and various other maxillofacial conditions [1, 20].

The software's processing time is contingent upon the machine's capabilities. If the machine fulfils the minimum hardware requirements, specifically possessing CUDA‐enabled graphics, the processing time will synchronise with the duration of the sample video. Notably, recent advancements include the introduction of dedicated AI cores within processors [21]. This development suggests that processing times are likely to experience a substantial decrease in the coming years, making it a subject for future exploration.

## 5 CONCLUSION

The research successfully applied landmark tracking using consumer‐grade camera footage, employing arbitrary width reference and fiducial marking methodologies. Both techniques yielded similar outcomes in tracking lateral excursions, with unique limitations. However, assessing midface and lower face attributes, the fiducial marker method exhibited greater variability. Regardless of the technique applied, hard tissue movement was typically seen to be 30% less than soft tissue among the participants. Additionally, a significant number of participants consistently displayed a 5–10° head tilt.

## AUTHOR CONTRIBUTIONS

**Farhan Hasin Saad**: Conceptualization; data curation; formal analysis; investigation; methodology; software; writing—original draft. **Taseef Hasan Farook**: Conceptualization; data curation; funding acquisition; project administration; resources; validation; writing—original draft; writing—review and editing. **Saif Ahmed**: Conceptualization; investigation; methodology; project administration; software; supervision. **Yang Zhao**: Methodology; project administration; software; validation; writing—review and editing. **Zhibin Liao**: Project administration; validation; visualization; writing—review and editing. **Johan W. Verjans**: Project administration; supervision; validation; writing—review and editing. **James Dudley**: Conceptualization; formal analysis; funding acquisition; project administration; resources; writing—original draft; writing—review and editing; supervision.

## CONFLICT OF INTEREST STATEMENT

The authors declare no conflicts of interest

## Data Availability

All data has been made available within the manuscript. The open‐source codes to the software are available through Github (https://github.com/ElsevierSoftwareX/SOFTX‐D‐23‐00353; Last accessed on 12 January 2024)
